# A case of methyl ethyl ketone peroxide poisoning and a review of complications and their management

**DOI:** 10.1186/s12995-015-0071-8

**Published:** 2015-07-31

**Authors:** Isurujith K. Liyanage, Mitrakrishnan R. Navinan, A C A Pathirana, H R I S Herath, Jevon Yudhishdran, Nilesh Fernandopulle, Aruna Kulatunga

**Affiliations:** National Hospital of Sri Lanka, Colombo 10, Sri Lanka; Department of Pharmacology, Faculty of Medical Sciences, University of Sri Jayewardenepura, Nugegoda, Sri Lanka

**Keywords:** Methyl Ethyl Ketone Peroxide, Management of corrosive gastrointestinal burns, Occupational safety

## Abstract

Methyl Ethyl Ketone Peroxide (MEKP) is a highly toxic clear liquid used as a solvent. It is a strong oxidizing agent and a corrosive. Acute and chronic toxicity can occur as an occupational hazard. Ingestion is associated with corrosive burns leading to stricture formation, inhalational pneumonitis, acidosis, liver failure and renal failure. In this paper we present a case of a young patient who intentionally ingested MEKP. The patient developed multiple complications including proximal intestinal obstruction, acidosis and acute kidney injury. He was managed conservatively and recovered after a prolonged hospital stay. He had multiple inflammatory strictures on esophageal endoscopy, which improved over 3–6 moths.

## Background

Methyl Ethyl Ketone Peroxide (MEKP) is used as a hardener in manufacture of resins, synthetic rubber and other petrochemical plastics. It is an ingredient of paints, varnishes and paint removers. MEKP is also used in fiberglass and plastics industry as a curing agent. It is an organic peroxide, which is explosive in its pure form. Hence it is commercially available as a 40-60 % solution with stabilizing agents such as dimethyl phthalate, cyclohexane peroxide, or diallyl phthalate [1, 2].

MEKP is listed as a highly toxic substance and is categorized into United Nations Hazard class 5.2. [3]. Its colorless nature and minimal odor has led to accidental ingestion both among adults and children [4, 5]. Several cases of intentional ingestion for self-harm or suicide have been reported [6]. In addition, poisoning by inhalation and spillage to eyes leading to corrosive damage has also been reported. Unprotected workers are victims of chronic exposure and toxicity [7].

Commercially available preparations of MEKP are strong oxidizing substances. They are known to produce alkylperoxyl radicals upon contact with metal ions, a process accelerated by the presence of iron in the heme molecule in biological systems. Tissue damage is believed to be caused by these free radicals, which denature organic molecules, including peroxidation of lipids. Further toxicity is caused by the acidity of the chemicals produced [8].

In a case report and review of literature, van Enckevort et. al described the clinical features of acute intoxication in four categories. These include inflammation and ulceration of exposed gastrointestinal tract, acute oxidative liver injury, complications of formation of organic acids including metabolic acidosis and secondary acute complications. Secondary complications include acute renal failure secondary to rhabdomyolysis, ventilator assisted pneumonia, myocarditis and acute respiratory distress syndrome [9].

Acute gastrointestinal (GI) injury has been observed in all documented case reports. It can lead to bleeding or perforation of the GI tract which is potentially fatal [10]. Severe pain due to chemical burns will develop soon after ingestion. The extent of injuries visible on mouth and oropharynx are known to be poor predictors of distal injuries. The presence of stridor, odynophagia (pain on swallowing), abdominal guarding and rigidity however, indicate the presence of distal GI injuries [11]. Long term problems such as esophageal strictures requiring multiple endoscopic dilatations has been reported [5]. Liver failure due to oxidative hepatocyte injury is the most significant single cause of mortality in the previous case reports [4, 9, 12, 13]. Reversible acute kidney injury (AKI) has been reported in almost a third of published case studies. This is mainly attributed to rhabdomyolysis and myoglobinuria [14]. MEKP can be aspirated resulting in proximal airway edema and obstruction, pneumonitis or acute respiratory distress syndrome [15]. Respiratory complications have been seen frequently (38 %) and has also been a direct cause for documented fatality [9].

### Case report

The patient was a 31-year-old healthy male who is self-employed in the shipping industry as a repairman of fiberglass boats. After consuming half a bottle of locally produced arrack (alcohol 38 % W/w) he ingested an estimated volume of 150 ml of MEKP containing solvent (60 % MEKP in phthalate solution) as an act of deliberate self-harm. He was unaware of the level of toxicity of the substance.

The patient developed severe throat and epigastric discomfort soon after ingestion. He was admitted to a regional hospital where he was given activated charcoal. A nasogastric tube was inserted in anticipation of erosive complications. Over the next 24 hours, his symptoms progressed with worsening irritation of the throat, development of abdominal pain, difficulty in breathing and noticeably reduced urine output. He was transferred to our tertiary care unit for further management of progressive multi organ involvement.

On general examination he was alert and coherent with Glasgow coma scale score of 15. There were multiple ulcerations in his mouth and oropharyngeal region. Nasogastric tube contents revealed coffee ground aspirate. Respiratory examination revealed rapid shallow breathing with a respiratory rate of 40 per minute. Auscultation of the lungs was unremarkable and oxygen saturation was 99 % on room air on pulse oxymetry. He was tachycardic (heart rate:104 beats per minute) with normal blood pressure and adequate peripheral perfusion. Severe guarding and tenderness over the epigastrium was present in examination of the abdomen. Bowel sounds were sluggish.

Preliminary investigations revealed progressively rising serum creatinine and blood urea. He had a neutrophil leukocytosis (Table 1). Arterial blood gas analysis showed a high anion gap metabolic acidosis with a pH of 7.21. Serum potassium was elevated at 6.4 mmol/L, which persisted through the first week of hospital stay. Ultrasound scan of the abdomen showed normal sized kidneys with evidence of acute renal failure. Subsequent complete blood counts showed a reducing trend in hemoglobin and platelets. Bite cells, Heinz bodies and fragmented red cells were also seen. Serum lactate dehydrogenase level was elevated. Direct Coombs test was negative. The reticulocyte count was 1.5 % showing an inadequate bone marrow response. Hemolysis settled by the second week of the illness.

**Table 1:** Basic investigations

	Reference range	D2	2nd week	3rd week	D28
Creatinine (umol/L)	60-120	366	706	800	253
Hemoglobin (g/dl)	-	12.2	4.4	7.5	12.1
Platelets 10^3^/mm^3^	150-450	49	87	170	245
White cell count 10^3^/mm^3^	4-11	12.9	22.2	5.3	8.2
AST (U/l)	10-35	611	212	24	29
ALT (U/l)	10-40	527	252	8	19
ALP (U/l)	100-360	105	143	129	-
Total Bilirubin (umol/L)	5-21	29	18	8	-
Direct Bilirubin	<3.4	25	14	5	
Urine RBC /hpf	-	>100		10 to 15	0
Urine pus cells /hpf	-	4 to 6		Field full	2 to 3
LDH (U/L)	140-280		812	643	343
Creatine Kinase (U/L)	25-174	457			

*AST* Aspartate transaminase, *ALT* alanine transaminase, *ALP* alkaline phosphatase, *RBC* red blood cells, *hpf* high power field, *LDH* Lactate dehydrogenase

The patient was managed in the ward. Acute management included intravenous sodium bicarbonate to correct acidosis and medical management of hyperkalemia. Urgent hemodialysis was followed by regular renal replacement therapy for the next four weeks. Repeated blood transfusions (8 units during the first week) were required to maintain hemoglobin levels.

**Fig. 1:**
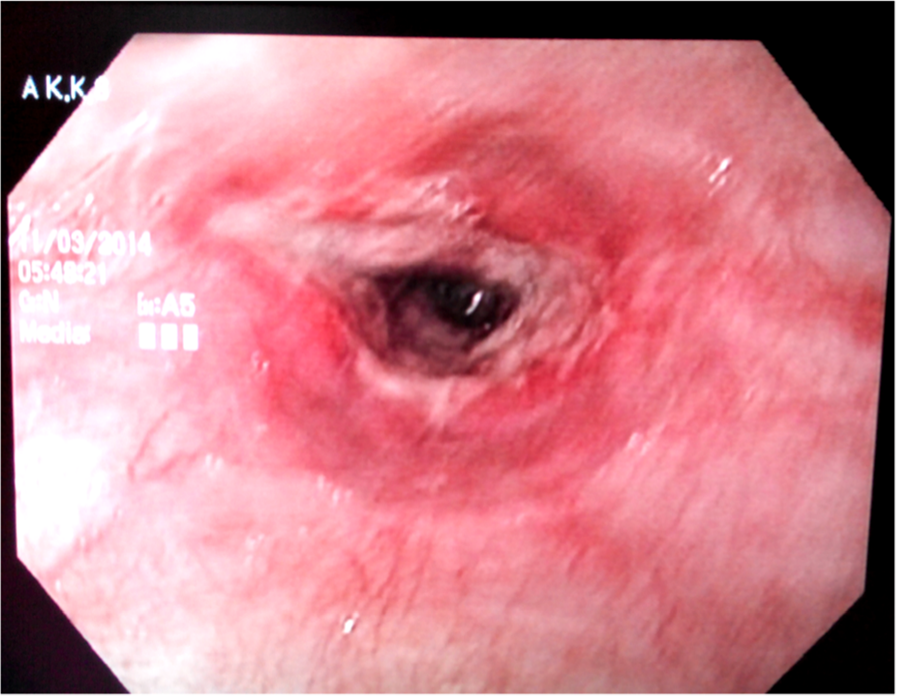
Fibrotic bands seen in upper gastrointestinal endoscopy on the 28^th^ day

Gastric protection was instituted with intravenous proton pump inhibitors. Parenteral feeding was initiated due to intolerance of oral feeds. On third day the patient developed large volume coffee ground aspirate through the NG tube, which later became bilious. This heralded distal duodenal obstruction, which was confirmed by a gastrografin study. This resolved after 2 weeks and liquid feeds were gradually started as tolerated. However, he had persistent odynophagia. Upper gastrointestinal endoscopy performed on day 28 revealed inflammatory lesions extending from upper esophagus to duodenum. There were slough, exudate and fibrotic bands but significant stricture formation was not seen (Fig. 1).

The patient was discharged on the 29^th^ day. He was tolerating semisolid diet despite having persisting odynophagia. He had lost 4.7 kg during his hospital stay. His follow up plan included a repeat endoscopic assessment in 3 months time, psychiatric assessment and follow up with the medical unit. He was also referred to the dietitian for nutritional support and supervision.

By his first review in 2 weeks the renal functions had returned to baseline. First follow up endoscopy at 3 months revealed slough, which was cleared by the passage of the endoscope. This resulted in temporary relief of odynophagia. However it recurred and the odynophagia persisted for six months. At 6-month review he was tolerating normal feeds with only slight discomfort. He has lost further 7 kg (weight on admission 68 kg) but features of other nutritional deficiencies were not present. One year after ingestion, he was asymptomatic and was gaining weight.

## Discussion

Lethal limit of MEKP is estimated to be 50–100 ml for adults (Lethal dose in rats: 65 mg/kg) [16]. The patient in this case study consumed a lethal dose of MEKP and developed most of the local and systemic complications of ingestion. Different management strategies have been tried in the past for acute poisoning. Almost all of these have been supportive until tissue damage and organ dysfunction recovers spontaneously.

MEKP is a potent oxidizing agent and antioxidant properties of vitamin E has been shown to be of benefit in rat models [17]. However there are no clinical data to support their use in acute poisoning [14].

Following ingestion of MEKP, the mouth should be rinsed with water. Drinking large volumes of milk or water to dilute the gastric contents has been practiced. It is advisable to continue of oral feeds as tolerated unless perforation is suspected [14]. Baseline investigations recommended are full blood count, renal functions, liver functions, arterial blood gas analysis, creatine kinase and coagulation profile. First line imaging includes plain X-ray films of the neck, chest and abdomen as clinically indicated. Intraluminal air shadows, air in the fascial planes, pleural effusions, and gas under diaphragm are X-ray features supporting gastrointestinal perforation [18]. Endoscopic evaluation is recommended within the first 24 hours of ingestion and may be beneficial up to 96 hours [19, 12]. After the initial 48–96 hours however, upper gastroesophageal endoscopy (UGE) should be avoided until after 2 weeks due to wound softening which can lead to perforation [11]. If perforation is suspected oral water soluble contrast studies (gastrografin) can be used to confirm and locate the site [18]. If perforated and severe continuous leakage of bowel contents is present surgical intervention may be needed.

This patient developed acute transient proximal intestinal obstruction, which spontaneously resolved over the first two weeks. This could have been due to mucosal edema due to the injuries. In the absence of esophageal perforation, insertion of nasogastric tube before the onset of edema was useful in managing this patient. It helped to identify obstruction when continuous bilious aspiration was noted. It also allowed continuation of enteral feeding when persistent odynophagia prevented the patient from tolerating oral feeds. Hence we recommend early insertion of a nasogastric tube within the first 24 hours unless severe edema or ulceration precludes its use. To prevent further GI injury, proton pump blockers should be used.

Delayed fibrosis and stricture formation leading to intestinal obstruction is one of the main long-term complications of corrosive ingestion as seen in this case of MEKP poisoning. This can occur up to one year after ingestion. This patient had multiple inflamed areas of sloughing in the first esophageal endoscopy, which indicated the possibility of long-term fibrosis and luminal narrowing. Endoscopic dilatation is needed to relieve the strictures if present. Further, endoscopic intra-lesional steroid injections are postulated to augment the affects of endoscopic dilatation and may reduce further fibrosis [13, 20]. However, evidence for use of steroid injections is minimal and recommendations vary widely. As the lesions were superficial, the gastroenterology team decided not to give intra-lesional steroid injections to our patient. Patients should be followed up after discharge to monitor for features of obstruction. In our patient, the follow up endoscopic examination itself cleared the inflammatory fibrotic bands producing dramatic improvement of his swallowing difficulty.

If swallowing difficulty is present, providing adequate long-term nutrition under the supervision of a specialist nutritionist is recommended. This patient had significant weight loss despite being on high protein diet until dysphagia and odynophagia improved. A weight gain was only noted after 9 to 12 months. If swallowing difficulty persists and impedes nutritional supplementation, endoscopic esophageal dilatation or esophageal resection is recommended [14].

Oxidative liver injury can be theoretically minimized or prevented by administration of N-acetyl cysteine (NAC) [21]. This should be administered within the first 24 hours of ingestion. One case study reports successful use of NAC as a liver protective agent [9]. However further evidence is needed to confirm its effectiveness.

Aspiration and other respiratory complications can be minimized by not inducing emesis. Aspiration if occurs should be managed symptomatically with supportive measures. Acute renal failure may necessitate repeated renal replacement therapy. There is minimal evidence to compare continuous renal replacement therapy with conventional dialysis and both methods have been used successfully [22].

Hematological complications such as bleeding, hemolysis and disseminated intravascular coagulation have been reported [9, 6]. Anemia in this patient was multifactorial, contributed by hemolysis, inadequate bone marrow response and gastrointestinal bleeding. Although continuing hemolysis was a possibility, coffee ground like NG aspirate and very low hemoglobin with symptoms of anemia warranted transfusions during the first weeks of treatment.

## Conclusions

MEKP is a potentially lethal industrial chemical. Acute poisoning can occur due to accidental or intentional ingestion. Ingestion can affect multiple organ systems, leading to short and long term morbidity. These include gastrointestinal burn injuries with stricture formation, acute liver failure, rhabdomyolysis, renal failure and metabolic acidosis. Management is mainly supportive. Careful monitoring for these complications with prompt intervention when they occur can save lives. Users of these chemicals may be unaware of the potential hazards. There is a need to increase the awareness on potential toxicity and safety measures among the handlers of these uncommon toxic substances.

## Consent

Informed written consent has been obtained from the patient for publication of this article and associated data and images.
